# Seed Rain, Soil Seed Bank, and Seedling Emergence Indicate Limited Potential for Self-Recovery in a Highly Disturbed, Tropical, Mixed Deciduous Forest

**DOI:** 10.3390/plants9101391

**Published:** 2020-10-20

**Authors:** Anussara Chalermsri, La-aw Ampornpan, Witoon Purahong

**Affiliations:** 1 Prasarnmit Demonstration School (Elementary), Faculty of Education, Srinakharinwirot University, Bangkok 10110, Thailand; anussarac@gmail.com; 2 Department of Biology, Faculty of Science, Srinakharinwirot University, Bangkok 10110, Thailand; la-aw@swu.ac.th; 3 Department of Soil Ecology, UFZ-Helmholtz Centre for Environmental Research, Theodor-Lieser-Str. 4, D-06120 Halle (Saale), Germany

**Keywords:** tropical forest, plant ecology, restoration, forest management, forest disturbance, seedling mortality

## Abstract

Human activity negatively affects the sustainability of forest ecosystems globally. Disturbed forests may or may not recover by themselves in a certain period of time. However, it is still unclear as to what parameters can be used to reasonably predict the potential for self-recovery of human-disturbed forests. Here, we combined seed rain, soil seed bank, and seed emergence experiments to evaluate the potential for self-recovery of a highly disturbed, tropical, mixed deciduous forest in northeastern Thailand. Our results show a limited potential for self-recovery of this forest due to low seedling input and storage and an extremely high mortality rate during the drought period. There were 15 tree species of seedlings present during the regeneration period in comparison with a total number of 56 tree species in current standing vegetation. During the dry season, only four tree seedling species survived, and the highest mortality rate reached 83.87%. We also found that the correspondence between the combined number of species and composition of plant communities obtained from seed rain, soil seed bank, and seedling emergence experiments and the standing vegetation was poor. We clearly show the temporal dynamics of the seed rain and seedling communities, which are driven by different plant reproductive phenology and dispersal mechanisms, and drought coupled with mortality. We conclude that this highly disturbed forest needs a management plan and could not recover by itself in a short period of time. We recommend the use of external seed and seedling supplies and the maintenance of soil water content (i.e., shading) during periods of drought in order to help increase seedling abundances and species richness, and to reduce the mortality rate.

## 1. Introduction

Human activity significantly affects the sustainability of forest ecosystems globally [1,2,3]. Deforestation and changes in land use intensity are common activities that could negatively impact native forest biota [3,4,5,6]. New seed input [7], buried seeds in the soil [8,9], resprouting [10], and seedling survival rates determine the development of subsequent vegetation after disturbances [11]. Thus, in pristine natural forests, continuous seed input and storage could ensure plant community regeneration following a disturbance [12]. In disturbed forests, especially in tropical forests, it is still unclear how these parameters change, and the regeneration of plant communities may depend on the intensity of disturbance [12,13]. The regeneration processes of tropical forests after a disturbance differ according to the type of disturbance (e.g., large, infrequent natural disturbances (hurricanes, floods, fire, etc.), burning, agriculture, forest clearing, or logging) [14,15]. Among these disturbances, logging is very common in tropical forests, and can significantly impact the forest structure and composition [14]. Logging removes forest cover, opening the way to secondary forest succession through regeneration of remnant vegetation, resprouting of roots and stems, and pioneer seedling establishment [10,14,16]. Colonization by seeds dispersed from outside the forest site is also possible at this stage [16]. Successional shifts in plant community composition occur over time, from light-demanding pioneer and early successional species toward late successional species [17].

In this study, human disturbances, including logging, burning, and agriculture, represent the main types of disturbances. The seed rain, soil seed bank, and seedling emergence and survival rates are crucial parameters in predicting the development of the plant community in a forest after disturbances [7,9,11]. Seedlings in forests can be obtained by the propagules recently deposited at the site, seed rain, or from propagules stored in the soil, the soil seed bank [18]. Seedling emergence and their survival rate together with the seed rain and soil seed bank determine the abundance and richness of new plants successfully established in forests [7,19]. Although these parameters are important, especially for evaluating the “self-recovery” of forests after disturbances, there are few studies that investigate these parameters together [20].

In this study, we aimed to evaluate the potential for the self-recovery of a highly disturbed, tropical, mixed deciduous forest in northeastern Thailand by investigating (i) the seed rain, (ii) the soil seed bank, and (iii) the seedling emergence and survival rates. Standing vegetation at the study site was used as the baseline for the plant community. We hypothesized that in this highly disturbed forest the seed rain and soil seed bank do not represent the total plant community and the seedling survival rate is low.

## 2. Results

### 2.1. Seed Rain: Low Seed Abundances Distributed to the Whole Study Area

**Figure 1:**
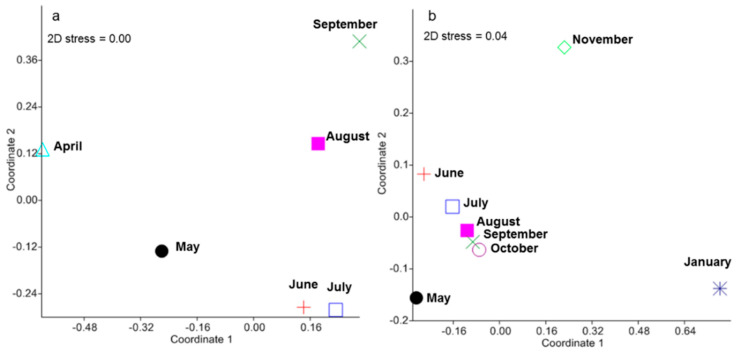
Non-metric multidimensional scaling (NMDS) of changes in the community composition of seed species (**a**) and tree seedling species (**b**) detected in different months.

A total of 1304 seeds belonging to 16 species (14 dicots and 2 monocots), 12 families, and 16 genera were detected in this experiment. These pools of plant species consisted of 3 herbaceous species (248 seeds) and 13 tree species (1056 seeds) (Table 1; Figure S1, supplementary materials). The average total and tree seed density values were 24.15 ± 2.60 and 19.57 ± 13.64 seed month^−1^ m^−2^, respectively. The locations of the seed traps (under the tree canopy, the bamboo patch, and the forest canopy gap) had a negligible effect on seed dispersal; seed richness ranged from 10 to 14 species (Table S1, supplementary materials). The highest detected taxa were *Gardenia sootepensis* (549 seeds, tree), *Chromolaena odoratum* (216 seeds, herbaceous), *Bauhinia malabarica* (165 seeds, tree), and *Lagerstroemia* sp. (120 seeds, tree) (Table 1; Figure S1, supplementary materials). Non-metric multidimensional scaling (NMDS) analysis showed strong changes in the composition of seed species detected in different months, except in June and July (Figure 1a).

### 2.2. Soil Seed Bank: Low Species Richness and Abundances of Tree Seed Storage

A total of 662 seedlings belonging to 35 species (28 dicots and 7 monocots), 15 families, and 21 genera germinated in the nursery from soil samples collected twice in the 50 subplots (Table S2, supplementary materials). The first soil sample had a much higher number of germinated seeds (595 vs. 67 seedlings, Table S2, supplementary materials) and greater species richness (all: 33 vs. 3 species, tree: 4 vs. 2) compared with the second sample. We detected low seed density among both the total group (331 ± 51.97 geminated seed m^−2^) and tree subgroup (48 ± 28.32 geminated seed m^−2^). *Asteraceae* and *Scrophulariaceae* were the most represented families, containing four and five detected species, respectively (Table S2, supplementary materials). In general, herbaceous species dominated the plant community in the soil seed bank. Two herbaceous species, *Hedyotis ovatifolia* and *Phyllanthus amarus*, accounted for 58.46% of the total plant abundance (Table S2, supplementary materials). We only detected five tree seedling species, all together accounting for 14.50% of the total plant abundance. Among tree seedlings, *Canariun subulatum* (10.42%) and *Lagerstroemia* (2.27%) are the most abundant, whereas *Croton roxburghii* was detected only once. The locations of the soil seed bank sample had no effect on the overall richness of tree seed storage at the two sampling times (germinated tree seed richness = 3 in all cases, Table S3, supplementary materials).

### 2.3. Tree Seedling Emergence and Survival Rates: Extremely Low Survival Rate in the Dry Season

**Figure 2:**
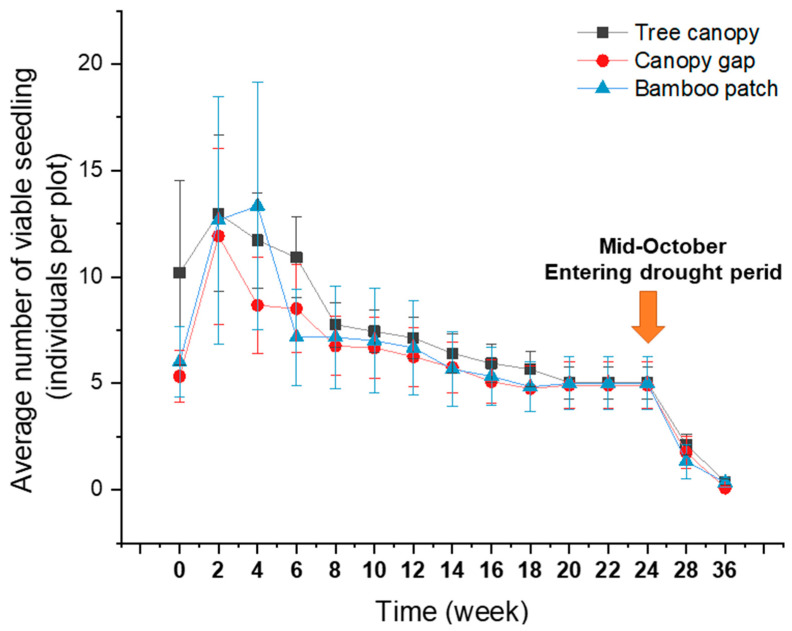
Number of viable seedlings (individuals per plot, mean ± standard error (SE)) in different plot types: the tree canopy, the canopy gap, and the bamboo patch.

**Figure 3:**
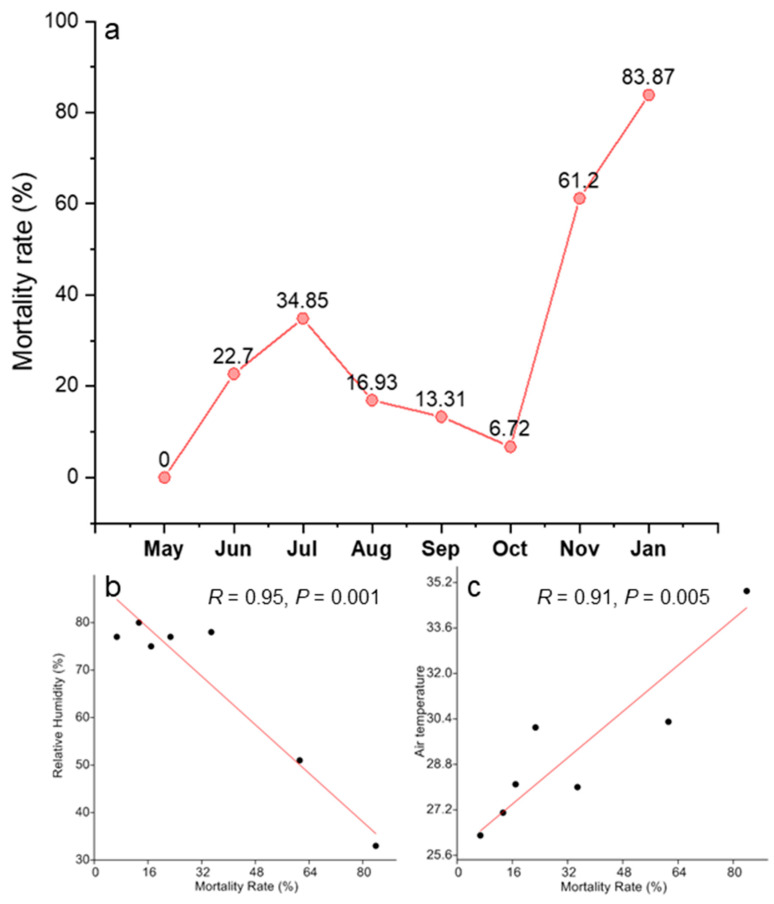
Mortality rate of tree seedlings across nine months in the seedling emergence experiment (**a**) and correlations between the mortality rate of tree seedlings and environmental factors: relative humidity (**b**) and air temperature (**c**).

Our seedling emergence experiment incorporated 727 individual tree seedlings distributed in 50 experimental plots. These seedlings belong to 15 tree species, 10 families, and 13 genera. *Canarium subulatu, Gardenia sootepensis*, and *Lagerstroemia* sp. highly dominated the seedling community, accounting for 84.18% of total abundance. We detected strong changes in the seedling community composition over time (Figure 1b). Changes in the number of viable seedlings across different sampling dates and locations are shown in Figure 2. At the beginning of the experiment in May, the seedling community was dominated by *Canarium subulatu* and *Gardenia sootepensis*. One month later, there was an increase in the abundance of *Lagerstroemia* sp. and the abundances of *Canarium subulatu* and *Gardenia sootepensis* had dropped. We also detected a newly emerging tree, *Suregada multiflorum*. These changes contributed to a strong shift in the seedling community composition, as shown in the NMDS ordination from May to June. During June to November, the seedling community was slightly altered due to a fluctuation in the mortality rate from low to moderate (Figure 2 and Figure 3). The extremely high mortality rate in November and January (the drought period) contributed to the strong shift in the seedling community composition during these two sampling times. In January, only four tree seedling species *Croton roxburghii*, *Gardenia sootepensis*, *Suregada multiflorum*, and *Memecylon edule* survived, and the mortality rate reached 83.87% (Figure 3). The locations of the subplots (tree canopy, bamboo patch, and forest canopy gap) had a negligible effect on the seedling mortality rate (Figure 2). We analyzed the factors correlated with the mortality rate of the tree seedlings in this experiment. Relative humidity showed a strong, negative correlation with the mortality rate (*R* = −0.95, *p* = 0.001), whereas air temperature showed a strong, positive correlation (*R* = 0.91, *p* = 0.005). Light intensity did not correlate with mortality rate (*R* = 0.06, *p* = 0.807). The correlations between mortality rate and all measured environmental factors are provided in Table S4 (supplementary materials). Partial correlation analysis showed that air temperature had a significant, positive correlation with mortality rate (*R* = 0.50, *p* = 0.035), whereas relative humidity and mortality rate showed no significant correlation (*R* = −0.31, *p* = 0.204) (Tables S5 and S6, supplementary materials).

### 2.4. Does the Combined Number of Species from the Three Methods Represent the Total Tree Species Pool at the Study Site?

**Figure 4:**
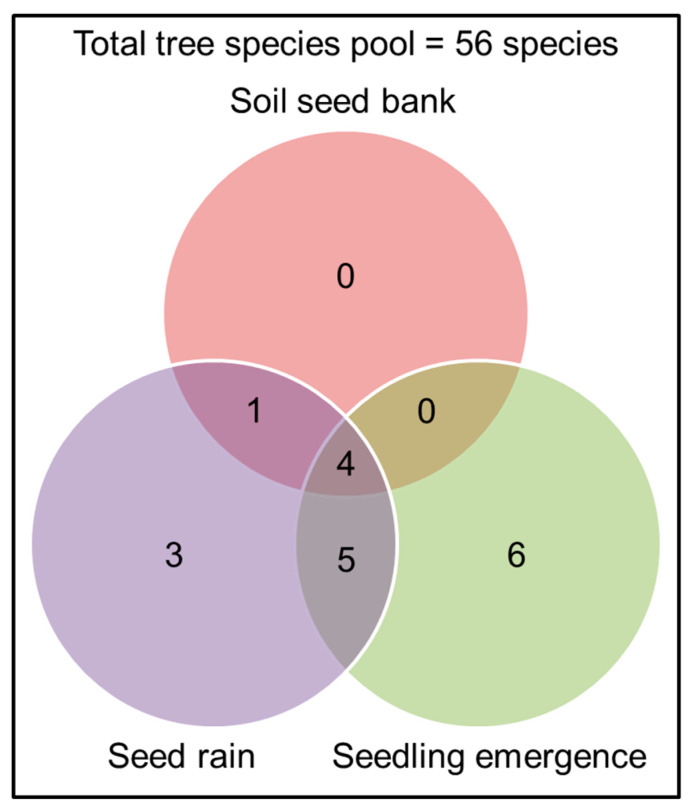
Number of tree species detected from the seed rain, soil seed bank, and seedling emergence experiment and total number of tree species in current standing vegetation (56 species).

Our results showed that in this highly disturbed forest, the tree species pool from the seed rain, soil seed bank, and seedling emergence experiments do not represent the total plant community (Figure 4). All detected tree species were subsets of the tree species pool in this study area. The seed rain and seedling emergence captured many more tree species than the soil seed bank (Table 1, Figure 4). All species present in the soil seed bank were found in the seed rain and seedling emergence, except *Irvingia malayana*. Three tree species were detected specifically from the seed rain and six tree species were detected specifically from the seedling emergence experiment (Table 1, Figure 4).

## 3. Discussion

### 3.1. Low Seed Input in Both Abundance and Richness

Our results on the seed rain indicate a low amount of seed input into this highly disturbed forest. The abundance and seed density values in this study are much lower than those obtained from other studies in primary and secondary tropical forests [7,13,21,22]. Furthermore, the species richness of the seeds that fell into the traps is also lower than that of other tropical forests [21,22,23]. Comparisons between the numbers of tree species detected in the tree canopy (forest stand) and the corresponding numbers of tree species detected in the seed rain, soil seed bank, and seedlings (and the percentage compared to the canopy) across different tropical forests are provided in Table S7 (supplementary materials). The number of tree species from the seed rain detected in this study (13 species) is also far below the richness of tree species pool (56 species) in this study area [24]. In this study, we found that the locations of the seed traps (under the tree canopy, the bamboo patch, and the canopy gap) had a negligible effect on seed dispersal, which may imply that the low abundance and low richness of the seed rain are likely due to the low abundance of mature trees, rather than the location effects. Human disturbances in this forest also strongly affect the plant community structure; more specifically, bamboo species are gaining dominance [25]. Bamboo-dominated areas are found to have lower seed rain rates across different regions [22]. Due to the relatively short sampling time for seed rain (6 months), we may not detect seeds from all mature plants due to a different plant reproductive phenology [26]. Nevertheless, our sampling times capture the peak period of seed rain in this study forest [25], and we may detect the remaining seeds of mature plants from the soil seed bank and seedling emergence experiments. Interestingly, we found that all species of trees detected in the seed rain are a subset of the tree species from the standing vegetation [24]; thus, there may be no, or very limited, long distance transportation of tree seeds (i.e., from other forests) to this forest.

### 3.2. Viable Seed Storage in Soil: Which Factors May Play an Important Role?

Similar to the seed rain, we also detected a low abundance and low richness of seeds stored in the soil (soil seed bank) of this highly disturbed forest compared with other tropical forests and degraded areas [12]. Low seed storage in tropical forests is not unexpected, as prompt germination seems to be the most common seed germination pattern [27]. However, extremely low seed storage in forest soil, as in this experiment, may result for other reasons. Our disturbed forest is also co-dominated by bamboo, which could significantly increase seed limitation events, including both limiting the number of seeds (source limitation) and limiting the dispersal of available seeds (dispersal limitation) [28]. The abundance of seed storage in forest soil is determined by complex seed bank dynamics through gains (i.e., plant seed production and seed dispersal) and losses (i.e., seed decay, seed death, and seed predation) [29]. Taking these seed gains into account, the highly disturbed forest has low seed production and disposal (as shown by the seed rain experiment). We conducted two sampling times at the same locations for the soil seed bank experiment to investigate the seed stored in the soil from previous years (first sample) and the current year (second sample). We found low amounts and low richness of seed storage in the forest soil, as compared with the standing vegetation, during both sampling times. Soil from the first sample has a much higher seed abundance and richness than the second sample, which contains only three plant species. This indicates the high loss of seeds from the current year’s production, and thus a small contribution to the overall soil seed bank.

### 3.3. Low Seedling Survival Rate: There Are Many Ways to Die

In tropical forests, seedling survival rates are negatively affected by multiple factors, including fire, drought, competition, predation, nutrient limitation, and plant pathogens [30,31,32]. Our results suggest that drought is the most important factor for the high mortality of seedlings. We observed the highest mortality rate (83.87%) in the drought period, which was characterized by extremely low rain fall and high temperatures. High mortality rates are consistently detected across different locations. There is a significant, positive correlation between air temperature and seedling mortality rate. Our results are consistent with other previous studies [32,33].

### 3.4. Seed Rain and Seedling Community Dynamics

We show clear temporal dynamics of the seed rain and seedling community in this highly disturbed forest. Different plant reproductive phenology and dispersal mechanisms drive the temporal pattern of the seed rain community as seeds in the forest ripen and disperse at different times [26]. In this forest, the peak time for seed rain is during the hot dry season (March to April), which is when we also detected the highest amount and highest species richness (85% of total tree species detected in the seed rain experiment) of seeds [25]. Seedling temporal patterns could be driven by seed input, climatic seasonality, soil fertility and moisture, natural and human disturbances, environmental factors related to germination, pattern of germination, and mortality rate [27,34,35]. In this study, we clearly show that drought coupled with mortality strongly shapes the pattern of the seedling community: seedling communities detected in November and January clearly separate from other sampling times on the NMDS ordination. In this study, we observed a mismatch between the combined number of species and the composition of plant communities obtained from the seed rain, soil seed bank, and seedling emergence experiments and that of the standing vegetation. This mismatch may result from the low seedling input and storage and the extremely high mortality rate in this highly disturbed forest. Another possibility is that in tropical forests, after a disturbance, there is common that pioneer species are replaced by late successional species [17]. Thus, the mismatch would depend on which state along this gradient the studied forest lies. Additionally, due to the limited temporal continuity of our experiment, we may not capture seeds from tree species with a different phenology [26].

## 4. Materials and Methods

### 4.1. Study Area

**Figure 5:**
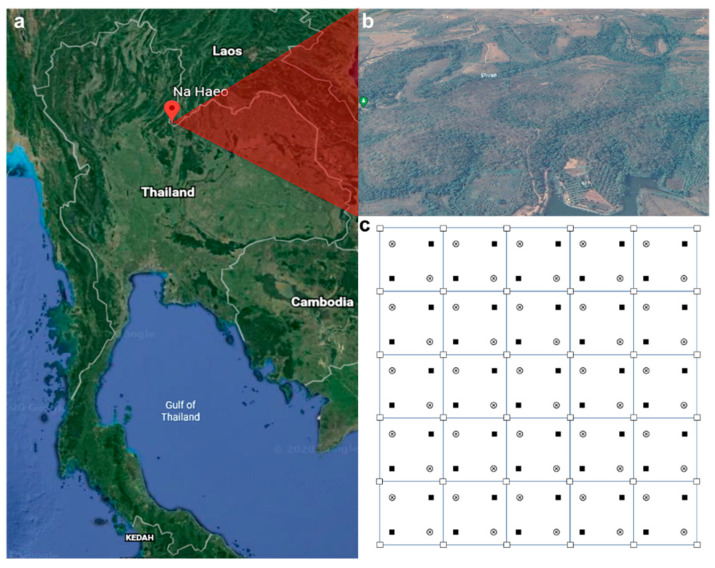
Satellite map of Thailand shows the location of the study site at Na Haeo district, Loei province, Thailand (**a**); Na Haeo Forest Reserve (17°29′ N, 101°04′ E) (**b**); and the 25 experimental plots (20 m × 20 m each, total area 10,000 m^2^) (**c**). The symbols □, ⊗, and ■ indicate 36 seed traps (0.5 m × 0.5 m), 50 subplots for soil sampling (20 cm × 20 cm × 5 cm), and 50 subplots for seedling emergence (1 m × 1 m), respectively.

**Figure 6:**
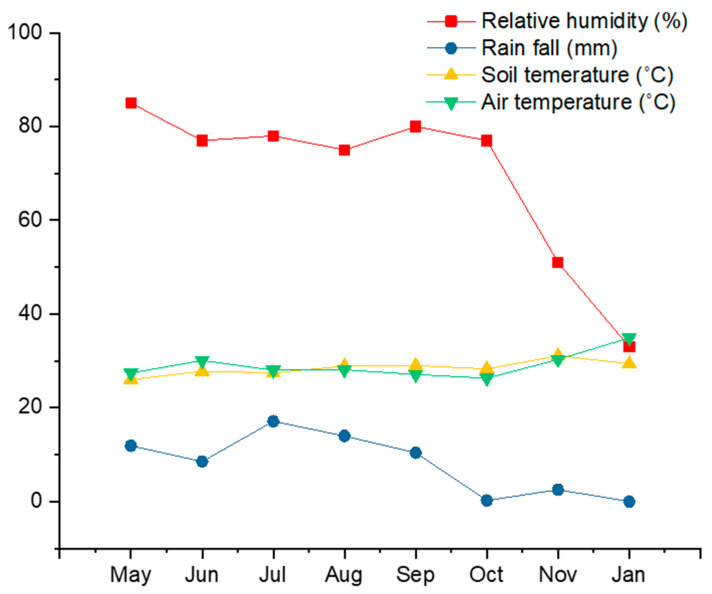
Average air and soil temperature, precipitation (rain fall), and relative humidity during the study period. Air temperature, relative humidity, and soil temperature were measured in all 25 plots every month from May 2004 to January 2005 (see experimental set up section). Rainfall was measured with a standard cylindrical rain gauge located nearby the experimental area (~1 km) at Srinakharinwirot University Research Station at Na Haeo.

The seed rain, soil seed bank, and seedling emergence and survival experiments were carried out in a mixed deciduous forest located in Na Haeo Forest Reserve (161 ha, 17°29′ N, 101°04′ E), Loei province, Thailand, as described previously (Figure 5) [23,25]. This forest has undergone different levels of human disturbances, including logging, burning, and agriculture, during the last century. Since then, the forest has been affected by continuous human disturbance. The forest composition and structure have been strongly modified from the original forest; more specifically, forest biomass and biodiversity have declined. The disturbance level of this forest is considered “high”, as this forest had an approximately 20% lower adult tree density (diameter at breast height > 4.5 cm) than the remnants of a protected original forest located at Phu Suan Sai (Na Haeo) National Park (1045 trees per ha). The total plant species richness was 131 species in the studied forest, as compared with 149 species in the protected original forest. The final aim of the restoration of this disturbed forest is the recovery of forest structure, biomass, biodiversity, and ecosystem functioning. The annual mean temperature and precipitation are 25 °C and 1551 mm, respectively. The elevation ranges from 400 to 600 m above sea level. The air and soil temperature, precipitation, and relative humidity during the study period (2004–2005) are shown in Figure 6. The dominant woody species in this forest are *Cananga latifolia*, *Lagerstroemia* sp., *Gardenia sootepensis*, *Spondiax laxiflora*, and *Pterocarpus macrocarpus*. Bamboo plants (*Gigantochloa albociliata*, *Bambusa tulda*, and *Cephalostachyum pergracile*) are also dominant in some parts of the forest, especially in highly disturbed areas [25]. Bamboo is an effective disturbance indicator in this study area [25]. Bamboo plants were almost entirely absent from the remnants of the protected original forest.

### 4.2. Experimental Setup

All three experiments were set up in a permanent forest site (10,000 m^2^), which was divided into 25 plots (400 m^2^ each) [24]. The total tree species in standing vegetation was determined in 2002 [24]. We counted adults (diameter at breast height > 4.5 cm) within the whole 400 m^2^ area in each of the 25 plots. In this forest, the majority of seeds from trees ripen and disperse during the dry season (March to April) and germinate during the wet season (starting in May). All experiments were established to correspond with these periods of time.

The seed rain was sampled from the end of March to September 2004 in 0.25 m^2^ (0.5 m × 0.5 m) seed traps composed of bamboo poles and frames equipped with calico fabric (<0.5 mm mesh) and suspended 80 cm above the ground to avoid seeds from herbaceous plants (Figure 5) [19]. Seed traps (36 traps in total) were set up at the corner of each plot and distributed across the forest to cover three different major areas: the tree canopy (17 traps), the bamboo patch (7 traps), and the forest canopy gap (12 traps). We collected and identified seeds from seed traps every week until the end of the experiment.

The soil seed bank experiment was carried out from April 2004 to January 2005. Two soil sampling campaigns were conducted at the same location in April and October 2004. The first soil sample was to investigate seed storage in soil from the previous year and the second soil sample was for the seed storage from the current year. We established two subplots within each of the 25 plots (400 m^2^ each), which produced 50 subplots (Figure 5). A soil sample was collected from each subplot (50 soil samples in total). These 50 soil samples were located in three different major areas: the tree canopy (29 samples), the bamboo patch (8 samples), and the forest gap (13 samples). For each sample, we collected a total of 2000 cm^3^ (20 cm × 20 cm × 5 cm) of soil [36]. Plant litter on the top of the soil layer, which may contain plant seeds, was also collected and pooled with each respective soil sample. All 50 samples were separately spread in trays with a surface area of 578 cm^2^ on top of clean sand. Ten additional trays filled with sand (without soil samples) were used as a control group. The trays were kept in a nursery (40% light transmission) equipped with fine mesh to avoid any external seed addition [37]. Sand used in this experiment was sieved (to remove any plant seeds and debris) and cleaned with water three times. The sand was poured into the trays, kept in a greenhouse, and watered every day for 2 weeks, and all germinated seeds were removed. At the end of the experiment, we found that no seeds had emerged in the control group. The first seedlings emerged during the first week, and emerging seedlings were recorded every day for the first month. Later, emerging seedlings were recorded every third day until no additional seedlings were found (42 weeks for the first soil sampling and 18 weeks for second soil sampling) [37]. The identified plant individuals were eliminated from the trays while the others were left either to grow in the original trays or were transplanted to other pots until they reached the blooming stage and could be identified [12].

The seedling emergence experiment was carried out for 9 months from May 2004 to January 2005. We established 2 subplots (1 m × 1 m) within the 25 plots (400 m^2^ each), which produced 50 subplots (Figure 5). These 50 subplots were located in three different major areas: the tree canopy (32 samples), the bamboo patch (6 samples) and the forest gap (12 samples). We collected data on seedling emergence (species and number) and seedling mortality (species and number) in each subplot every 2 weeks from May to October 2004, and later every month from November 2004 to January 2005. All seedlings were marked and checked. We also collected data on relative humidity, soil and air temperature, and light intensity every month from May 2004 to January 2005 (Figure 6, Table S8, supplementary materials). Soil and air temperatures were measured using a lab digital thermometer (model 9840, Taylor Precision Products, USA), which can measure temperatures ranging from −40 to 150 °C (resolution = 0.1 °C). Relative humidity was measured using a thermo-hygrometer (Oregon Scientific, USA, resolution = 1%, accuracy ± 1%). Light intensity was measured using a lux meter (LX-50, DIGICON, Thailand, accuracy ± 2%, 2000–50,000 lux). Rainfall was measured with a standard cylindrical rain gauge (diameter = 20 cm) located nearby the experimental area (~1 km) at Srinakharinwirot University Research Station at Na Haeo.

### 4.3. Statistical Analysis

Seed density in the seed rain experiment was analyzed as the total number of seeds collected from a seed trap and is reported as seed month^−1^ m^−2^. Seed density in the soil seed bank experiment was analyzed as the total number of germinated seeds collected from each soil sample and is reported as geminated seed m^−2^. Changes to the community composition of seed species and tree seedling species detected in different months were analyzed using non-metric multidimensional scaling (NMDS) based on relative abundance data and the Bray-Curtis distance measure implemented in PAST (PAleontological STatistics) [38]. Factors correlated with the mortality rate of tree seedlings in this experiment were analyzed using the Pearson product-moment correlation in SPSS (version 24). All datasets were tested for normality using the Jarque–Bera test. We checked for the correlations between different environmental factors and found that they were highly correlated (Table S4, supplementary materials). We used a partial correlation to analyze the relationships between (i) mortality rate and air temperature (rain fall, soil temperature, and relative humidity were used as control variables) and (ii) mortality rate and relative humidity (rain fall, soil temperature, and air temperature were used as control variables). Partial correlation was analyzed using SPSS.

## 5. Conclusions

In the present study, we combined seed rain, soil seed bank, and seed emergence experiments to evaluate the potential for self-recovery of a highly disturbed, tropical, mixed deciduous forest in northeastern Thailand. Our work provides evidence that there is limited potential for self-recovery of this forest due to low seedling input and storage and an extremely high mortality rate during periods of drought. The under-representation of the plant communities obtained from the seed rain, soil seed bank, and seedling emergence experiments compared with that of the standing vegetation community also confirms that this highly disturbed forest is unlikely to recover by itself in a short period of time. Thus, a forest management plan is needed. For restoration of this highly disturbed forest, we recommend the use of external seed and seedling supplies [39] and the maintenance of soil water content (i.e., shading) during periods of drought in order to help increase seedling abundances and species richness and reduce the mortality rate. The predicted changes in climate, especially in terms of the increase in temperatures and the decline in annual precipitation in Thailand [40], suggest that natural regeneration in such conditions will be even more difficult, if not impossible, because of the risk of the seedling mortality rate reaching 100%.

## Figures and Tables

**Table 1:** Abundances of tree seeds or seedlings obtained from three different methods: seed rain, soil seed bank (two repetitions), and seedling emergence. Two varieties of *Aporosa octandra* were treated as two different species in this study.

Tree Species	Seed Rain	Seed Bank (1)	Seed Bank (2)	Seedling Emergence
*Aporosa octandra* (B.-H. ex D.Don) Vick. var. *octandra*	-	-	-	2
*Aporosa octandra* (B.-H. ex D.Don) Vick. var. *yunnanensis*	-	-	-	8
*Bauhinia malabarica* Roxb.	165	-	-	-
*Bombax anceps* Pierre var. *anceps*	-	-	-	2
*Canarium subulatum* Guill.	86	7	62	214
*Cratoxylum formosum* (Jack) Dyer	5	-	-	26
*Croton roxburghii* N.P. Balakr.	6	1	-	35
*Dalbergia* sp.	1	-	-	-
*Gardenia sootepensis* Hutch.	549	7	-	210
*Garuga pinnata* Roxb.	43	-	-	2
*Irvingia malayana* Oliv. ex A.W.Benn	3	-	4	-
*Lagerstroemia* (*aff. venusta* Wall. ex Cl.)	120	15	-	188
*Memecylon edule* Roxb.	17	-	-	24
*Microcos tomentosa Sm.*	-	-	-	2
*Pterocarpus macrocarpus Kurz*	58	-	-	4
*Shorea roxburghii G.Don*	1	-	-	-
*Suregada multiflora (A. Juss.) Baill.*	-	-	-	6
*Terminalia chebula Retz. var. chebula*	-	-	-	1
*Terminalia triptera Stapf.*	2	-	-	3
Sum	1056	30	66	727

## Supplementary Materials

The following are available online at https://www.mdpi.com/2223-7747/9/10/1391/s1: **Figure S1**: Abundances of seeds from 16 plant species detected in this experiment; **Table S1**: Abundances of seeds from 16 plant species detected in the seed trap at different locations; **Table S2**: Abundances of seedlings from 35 plant species detected in the soil seed bank; **Table S3**: Plant abundances and species richness detected in the soil seed bank experiment; **Table S4**: Correlations among different factors tested in this study; **Table S5**: Partial correlations between mortality rate and air temperature; **Table S6:** Partial correlations between mortality rate and relative humidity; **Table S7:** Comparisons of the number of tree species detected in the tree canopy (forest stand) and corresponding number of tree species detected in the seed rain/soils seed bank/seedling (and the percentage compared to the canopy) across different tropical forests; and **Table S8**: Light intensity measured in this study during May 2004 to January 2005.

## References

[1] Sanderson E.W., Jaiteh M., Levy M.A., Redford K.H., Wannebo A.V., Woolmer G. (2002). The human footprint and the last of the wild. BioScience.

[2] Seppälä R. (2004). The future of forest research in a changing world. J. For. Res..

[3] Turner B.L., Lambin E.F., Reenberg A. (2007). The emergence of land change science for global environmental change and sustainability. Proc. Natl. Acad. Sci. USA.

[4] Gibson L., Lee T.M., Koh L.P., Brook B.W., Gardner T.A., Barlow J., Peres C.A., Bradshaw C.J.A., Laurance W.F., Lovejoy T.E. (2011). Primary forests are irreplaceable for sustaining tropical biodiversity. Nature.

[5] Purahong W., Kahl T., Schloter M., Bauhus J., Buscot F., Krüger D. (2014). Comparing fungal richness and community composition in coarse woody debris in Central European beech forests under three types of management. Mycol. Prog..

[6] Purahong W., Hoppe B., Kahl T., Schloter M., Schulze E.-D., Bauhus J., Buscot F., Krüger D. (2014). Changes within a single land-use category alter microbial diversity and community structure: Molecular evidence from wood-inhabiting fungi in forest ecosystems. J. Environ. Manag..

[7] Reid J.L., Holl K.D., Zahawi R.A. (2015). Seed dispersal limitations shift over time in tropical forest restoration. Ecol. Appl..

[8] Sakai A., Sato S., Sakai T., Kuramoto S., Tabuchi R. (2005). A soil seed bank in a mature conifer plantation and establishment of seedlings after clear-cutting in southwest Japan. J. For. Res..

[9] Tamura A. (2016). Potential of soil seed banks in the ecological restoration of overgrazed floor vegetation in a cool-temperate old-growth damp forest in eastern Japan. J. For. Res..

[10] Schafer J.L., Just M.G. (2014). Size dependency of post-disturbance recovery of multi-stemmed resprouting trees. PLoS ONE.

[11] Guariguata M.R., Ostertag R. (2001). Neotropical secondary forest succession: Changes in structural and functional characteristics. For. Ecol. Manag..

[12] Tekle K., Bekele T. (2000). The role of soil seed banks in the rehabilitation of degraded hillslopes in Southern Wello, Ethiopia. Biotropica.

[13] Holl K.D., Loik M.E., Lin E.H.V., Samuels I.A. (2000). Tropical montane forest restoration in Costa Rica: Overcoming barriers to dispersal and establishment. Restor. Ecol..

[14] Chazdon R.L. (2003). Tropical forest recovery: Legacies of human impact and natural disturbances. Perspect. Plant Ecol. Evol. Syst..

[15] Cole L.E.S., Bhagwat S.A., Willis K.J. (2014). Recovery and resilience of tropical forests after disturbance. Nat. Commun..

[16] Holl K.D. (2013). Restoring tropical forest. Nat. Educ. Knowl..

[17] Chambers J.Q., Negron-Juarez R.I., Marra D.M., Di Vittorio A., Tews J., Roberts D., Ribeiro G.H.P.M., Trumbore S.E., Higuchi N. (2013). The steady-state mosaic of disturbance and succession across an old-growth Central Amazon forest landscape. Proc. Natl. Acad. Sci. USA.

[18] Moles A.T., Drake D.R. (1999). Potential contributions of the seed rain and seed bank to regeneration of native forest under plantation pine in New Zealand. N. Z. J. Bot..

[19] Catovsky S., Bazzaz F.A. (2000). The role of resource interactions and seedling regeneration in maintaining a positive feedback in hemlock stands. J. Ecol..

[20] Du X., Guo Q., Gao X., Ma K. (2007). Seed rain, soil seed bank, seed loss and regeneration of Castanopsis fargesii (Fagaceae) in a subtropical evergreen broad-leaved forest. For. Ecol. Manag..

[21] Ceccon E., Hernández P. (2009). Seed rain dynamics following disturbance exclusion in a secondary tropical dry forest in Morelos, Mexico. Rev. Biol. Trop..

[22] Grombone-Guaratini M.T., Alves L.F., Vinha D., Franco G.A.D.C. (2014). Seed rain in areas with and without bamboo dominance within an urban fragment of the Atlantic Forest. Acta Bot. Bras..

[23] Martinez-Ramos M., Soto-Castro A. (1993). Seed rain and advanced regeneration in a tropical rain forest. Vegetatio.

[24] Dhillion S., Ampornpan L., Austreng I. (2003). Land Use and Plant Diversity in Ban Bung and Na Haeo Forest Reserve.

[25] Larpkern P., Moe S.R., Totland Ø. (2011). Bamboo dominance reduces tree regeneration in a disturbed tropical forest. Oecologia.

[26] Sakai S., Momose K., Yumoto T., Nagamitsu T., Nagamasu H., Hamid A.A., Nakashizuka T. (1999). Plant reproductive phenology over four years including an episode of general flowering in a lowland dipterocarp forest, Sarawak, Malaysia. Am. J. Bot..

[27] Vázquez-Yanes C., Orozco-Segovia A. (1993). Patterns of seed longevity and germination in the tropical rainforest. Annu. Rev. Ecol. Syst..

[28] Rother D.C., Rodrigues R.R., Pizo M.A. (2009). Effects of bamboo stands on seed rain and seed limitation in a rainforest. For. Ecol. Manag..

[29] Olano J.M., Caballero I., Escudero A. (2012). Soil seed bank recovery occurs more rapidly than expected in semi-arid Mediterranean gypsum vegetation. Ann. Bot..

[30] Gallery R.E., Moore D.J.P., Dalling J.W. (2010). Interspecific variation in susceptibility to fungal pathogens in seeds of 10 tree species in the neotropical genus Cecropia. J. Ecol..

[31] Salinas-Peba L., Parra-Tabla V., Campo J., Munguía-Rosas M.A. (2013). Survival and growth of dominant tree seedlings in seasonally tropical dry forests of Yucatan: Site and fertilization effects. J. Plant Ecol..

[32] Cardoso A.W., Medina-Vega J.A., Malhi Y., Adu-Bredu S., Ametsitsi G.K.D., Djagbletey G., van Langevelde F., Veenendaal E., Oliveras I. (2016). Winners and losers: Tropical forest tree seedling survival across a West African forest–savanna transition. Ecol. Evol..

[33] Engelbrecht B.M.J., Kursar T.A., Tyree M.T. (2005). Drought effects on seedling survival in a tropical moist forest. Trees.

[34] Norden N., Chave J., Caubère A., Châtelet P., Ferroni N., Forget P.-M., Thébaud C. (2007). Is temporal variation of seedling communities determined by environment or by seed arrival? A test in a neotropical forest. J. Ecol..

[35] Ceccon E., Huante P., Rincón E. (2006). Abiotic factors influencing tropical dry forests regeneration. Braz. Arch. Biol. Technol..

[36] Singhakumara B.M.P., Uduporuwa R.S.J.P., Ashton P.M.S. (2000). Soil seed banks in relation to light and topographic position of a hill dipterocarp forest in Sri Lanka1. Biotropica.

[37] Butler B.J., Chazdon R.L. (1998). Species richness, spatial variation, and abundance of the soil seed bank of a secondary tropical rain forest1. Biotropica.

[38] Hammer Ø., Harper D.A.T., Ryan P.D. (2001). PAST: Paleontological statistics software package for education and data analysis. Palaeontol. Electron..

[39] Waiboonya P., Elliott S. (2020). Sowing time and direct seeding success of native tree species for restoring tropical forest ecosystems in northern Thailand. New For..

[40] Marks D. (2011). Climate change and Thailand: Impact and response. Contemp. Southeast Asia.

