# Supplementary material for: Seed Rain, Soil Seed Bank, and Seedling Emergence Indicate Limited Potential for Self-Recovery in a Highly Disturbed, Tropical, Mixed Deciduous Forest
Source: Plants (Basel). 2020 Oct 20;9(10):1391. doi: 10.3390/plants9101391 (PMC7589040; doi:10.3390/plants9101391)

Supplementary materials

Seed rain, soil seed bank and seedling emergence indicate limited potential for self-recovery in a highly disturbed tropical mixed deciduous forest

Anussara Chalermsri[1, 2], La-aw Ampornpan[2], Witoon Purahong[3]

[1]Prasarnmit demonstration school (Elementary), Faculty of Education, Srinakharinwirot University, Bangkok, 10110, Thailand, [2]Department of Biology, Faculty of Science, Srinakharinwirot University, Bangkok, 10110, Thailand, [3]UFZ-Helmholtz Centre for Environmental Research, Department of Soil Ecology, Theodor-Lieser-Str. 4, D-06120 Halle (Saale), Germany.

Table S1. Abundances of seeds from 16 plant species detected in the seed trap at different locations (under tree canopy, bamboo patch and canopy gap).

Plant species	Tree canopy	Bamboo patch	Canopy gap
Bauhinia malabarica Roxb.	1	-	-
Canarium subulatum Guill.	5	3	1
Chromolaena odoratum (L.) R.M.King & H.Rob.	11	6	10
Cratoxylum formosum (Jack) Dyer	1	1	-
Croton roxburghii N.P. Balakr.	1	-	1
Dalbergia sp.	-	1	-
Gardenia sootepensis Hutch.	5	2	4
Garuga pinnata Roxb.	4	1	1
Imperata cylindrica (L.) P.Beauv.	11	2	5
Irvingia malayana Oliv.ex A.W.Benn	3	2	-
Lagerstroemia (*aff. venusta* Wall. ex Cl.)	13	6	8
Memecylon edule Roxb.	1	-	1
Pennisetum polystachyon Schult	3	3	2
Pterocarpus macrocarpus Kurz	7	5	5
Shorea roxburghii G.Don	-	1	-
Terminalia triptera Stapf	2	-	-

Table S2 Abundances of seedling from 35 plant species detected in soil seed bank (2 sampling times). (C = Climbing plant, H = Herbaceous plant, T = tree, Mono = monocots, Dicot = monocots).

Plant species	Family	Plant group	Soil seed bank (1)	Soil seed bank (2)	Total
Blumea sp. 1	Asteraceae	Dicot (H)	2	0	2
Blumea sp. 2	Asteraceae	Dicot (H)	3	0	3
Canariun subulatum Guill.	Burseraceae	Dicot (T)	7	62	69
Chromolaena odoratum (L.) R.M.King&H.Rob	Asteraceae	Dicot (H)	15	0	15
Conyza sp.	Asteraceae	Dicot (H)	3	0	3
Croton roxburghii N.P. Balakr.	Euphorbiaceae	Dicot (T)	1	0	1
Cyperus sp. 1	Cyperaceae	Monocot (H)	5	0	5
Cyperus sp. 2	Cyperaceae	Monocot (H)	19	0	19
Cyrtococcum accrescens (Trin.) Stapf	Poaceae	Monocot (H)	8	0	8
Dioscorea sp.	Dioscoreaceae	Dicot (C)	1	0	1
Entada rheedii Spreng. *	Fabaceae	Dicot (C)	0	1	1
Gardenia sootepensis Hutch	Rubiaceae	Dicot (T)	7	0	7
Harrisonia perforata (Blanco) Merr.	Simaroubaceae	Dicot (C)	1	0	1
Hedyotis ovatifolia Cav.	Rubiaceae	Dicot (H)	298	0	298
Imperata cylindrica (L.) P.Beauv.	Poaceae	Dicot (H)	2	0	2
Irvingia malayana Oliv. ex A.W.Benn *	Irvingiaceae	Dicot (T)	0	4	4
Lagerstroemia (*aff. venusta* Wall. ex Cl.)	Lythraceae	Dicot (T)	15	0	15
Lindernia ciliata (Colsm.) Pennell	Scrophulariaceae	Dicot (H)	26	0	26
Lindernia crustacea (L.) F.Muell. var. *crustacea*	Scrophulariaceae	Dicot (H)	20	0	20
Lindernia sp. 1	Scrophulariaceae	Dicot (H)	16	0	16
Lindernia sp. 2	Scrophulariaceae	Dicot (H)	14	0	14
Melastoma sp.	Melastomataceae	Dicot (H)	1	0	1
Murdannia sp.	Commelianaceae	Monocot (H)	5	0	5
Phyllanthus amarus Schumach.&Thonn.	Euphorbiaceae	Dicot (H)	89	0	89

**Plant species**	**Family**	**Plant group**	**Soil seed bank (1)**	**Soil seed bank (2)**	**Total**
Torenia flava Ham. ex Benth	Scrophulariaceae	Dicot (H)	14	0	14
Triumfetta sp.	Tiliaceae	Dicot (H)	2	0	2
unidentified	unidentified	Monocot (H)	1	0	1
unidentified	unidentified	Dicot (H)	1	0	1
unidentified	unidentified	Dicot (H)	1	0	1
unidentified	unidentified	Monocot (H)	8	0	8
unidentified	unidentified	Dicot (H)	1	0	1
unidentified	unidentified	Dicot (H)	2	0	2
unidentified	unidentified	Monocot (H)	1	0	1
unidentified	unidentified	Dicot (H)	5	0	5
unidentified	unidentified	Dicot (H)	1	0	1
Sum			595	67	662
Density			297.5	33.5	331
SD			51.3	34.39	51.97

Table S3 Plant abundances and species richness detected in soil seed bank experiment.

**Sampling Locations**	**First soil seed bank sampling**		**Second soil seed bank sampling**	
	**Number of tree seedling species (abundance)**	**Number of herbaceous seedling species (abundance)**	**Number of tree seedling species (abundance)**	**Number of herbaceous seedling species (abundance)**
Tree canopy	3 (19)	25 (250)	1 (54)*	-
Bamboo patch	1 (1)	14 (102)	2 (8)	-
Canopy gap	2 (10)	19 (213)	2 (4)*	1 (1)
Sum	4 (30)	29 (565)	2 (66)	1 (1)

*One tree seedling species from the second soil seed bank sampling overlapped with the first soil seed bank sampling.

Table S4. Correlations among different factors tested in this study. Bold indicates significant values ($P < 0.05$).

Correlations

		Mortality rate (%)	Relative humidity (%)	Rain fall (mm)	Soil temerature (˚C)	Air temperature (˚C)	Light intensity (x1,000 lux)
Mortality rate (%)	Pearson Correlation	1					
	Sig. (2-tailed)						
Relative humidity (%)	Pearson Correlation	**-.917****	1				
	Sig. (2-tailed)	.000					
Rain fall (mm)	Pearson Correlation	**-.440***	**.632****	1			
	Sig. (2-tailed)	.046	.002				
Soil temperature (˚C)	Pearson Correlation	**.571****	**-.637****	**-.515***	1		
	Sig. (2-tailed)	.007	.002	.017			
Air temperature (˚C)	Pearson Correlation	**.907****	**-.896****	-.432	.362	1	
	Sig. (2-tailed)	.000	.000	.051	.107		
Light intensity (x1,000 lux)	Pearson Correlation	.057	.063	.390	**-.703****	.195	1
	Sig. (2-tailed)	.807	.785	.081	.000	.397	

**. Correlation is significant at the 0.01 level (2-tailed).

*. Correlation is significant at the 0.05 level (2-tailed).

Table S5. Partial correlations between mortality rate and air temperature (rain fall, soil temperature and relative humidity were used as control variables). Bold indicates significant values ($P < 0.05$).

Correlations

Control Variables			Mortality rate	Air temperature	Rain fall	Soil temperature	Relative humidity
-none-[a]	Mortality rate	Correlation	1.000				
		Significance (2-tailed)	.				
	Air temperature	Correlation	**.907**	1.000			
		Significance (2-tailed)	.000	.			
	Rain fall	Correlation	**-.440**	-.432	1.000		
		Significance (2-tailed)	.046	.051	.		
	Soil temperature	Correlation	**.571**	.362	**-.515**	1.000	
		Significance (2-tailed)	.007	.107	.017	.	
	Relative humidity	Correlation	**-.917**	**-.896**	**.632**	**-.637**	1.000
		Significance (2-tailed)	.000	.000	.002	.002	.
Rain fall & Soil temperature & Relative humidity	Mortality rate	Correlation	1.000	**.498**			
		Significance (2-tailed)	.	.035			

Table S6 Partial correlations between mortality rate and relative humidity (rain fall, soil temperature and air temperature were used as control variables). Bold indicates significant values (P < 0.05).

Correlations

Control Variables			Mortality rate	Relative humidity	Rain fall	Soil temperature	Air temperature
-none-[a]	Mortality rate	Correlation	1.000				
		Significance (2-tailed)	.				
	Relative humidity	Correlation	**-.917**	1.000			
		Significance (2-tailed)	.000	.			
	Rain fall	Correlation	**-.440**	**.632**	1.000		
		Significance (2-tailed)	.046	.002	.		
	Soil temperature	Correlation	**.571**	**-.637**	**-.515**	1.000	
		Significance (2-tailed)	.007	.002	.017	.	
	Air temperature	Correlation	**.907**	**-.896**	-.432	.362	1.000
		Significance (2-tailed)	.000	.000	.051	.107	.
Rain fall & Soil temperature & Air temperature	Mortality rate	Correlation	1.000	-.314			
		Significance (2-tailed)	.	.204			

Table S7 Comparisons of number of tree species detected in tree canopy (forest stand) and corresponding number of tree species detected as seed rain/soils seed bank/seedling (and the percentage compared to the canopy) across different tropical forests.

Forest type and location	Type of disturbance	Number of species in the canopy/total tree species pool (size, density)	Number of species in seed rain /soils seed bank/seedling (and the percentage compared to the total tree species pool)	Tree seed abundance (seeds $month^{-1} m^{-2}$)	Reference
Tropical Mixed Deciduous Forest, Thailand	Human disturbances (including logging, burning and agriculture)	56 species (1 ha, 841 individuals per ha.)	Seed rain: 13 (23.2%)<br>Soils seed bank: 5 (8.9%)<br>Seedling: 15 (26.8%)	19.57	This study.
Tropical deciduous forest, Mexico	Human disturbances (including logging, burning and agriculture)	40* species (1 ha, 1425 individuals per ha.)	Seed rain: 18 (57.5%)*	33.33*	[1]
Tropical secondary forest, Brazil	Human disturbances (including logging and burning)	N.A. (0.7 ha, 1767 individuals per ha.)	Seed rain: 25 (N.A.)<br>Soils seed bank: 12 (N.A.)	26.43	[2]
Tropical secondary forest dominated by bamboo (*Aulonemia aristulata*), Brazil	Human disturbances (including logging and burning)	N.A. (0.7 ha, 1354 individuals per ha.)	Seed rain: 26 (N.A.)<br>Soils seed bank: 16 (N.A.)	21.88	[2]
Tropical premontane humid forest, Costa Rica	Human disturbances (including forest clearing and agriculture)	N.A.	N.A.	89.17	[3]
Northernmost neotropical rain forest, Mexico (Plot 1, patch age >35 years)	Preserve area	31 species (0.063 ha, 1104 individuals per ha.)	Seed rain (all tree species): 36 (116.1%)<br>Seed rain (local tree species): 8 (25.8%)<br><br>Recruited-seedling (all tree species): 15 (48.4%)<br>Recruited-seedling (local tree species): 11 (35.5%)	N.A.	[4]
Northernmost neotropical rain forest, Mexico (Plot 2, patch age >35 years)	Preserve area	33 species (0.063 ha, 960 individuals per ha.)	Seed rain (all tree species): 34 (103%)<br>Seed rain (local tree species): 15 (45.5%)<br><br>Recruited-seedling (all tree species): 15 (48.4%)<br>Recruited-seedling (local tree species): 8 (24.2%)	N.A.	[4]
Northernmost neotropical rain forest, Mexico (Plot 3, patch age 20 years)	Preserve area	30 species (0.063 ha, 992 individuals per ha.)	Seed rain (all tree species): 34 (113%)<br>Seed rain (local tree species): 15 (50%)	N.A.	[4]

Forest type and location	Type of disturbance	Number of species in the canopy/total tree species pool (size, density)	Number of species in seed rain /soils seed bank/seedling (and the percentage compared to the total tree species pool)	Tree seed abundance (seeds $month^{-1} m^{-2}$)	Reference
			Recruited-seedling (all tree species): 15 (50%)<br>Recruited-seedling (local tree species): 7 (23.3%)		
Northernmost neotropical rain forest, Mexico (Plot 4, patch age >35 years)	Preserve area	25 species (0.063 ha, 816 individuals per ha.)	Seed rain (all tree species): 33 (132%)<br>Seed rain (local tree species): 16 (64%)<br><br>Recruited-seedling (all tree species): 19 (76%)<br>Recruited-seedling (local tree species): 10 (40%)	N.A.	[4]
Northernmost neotropical rain forest, Mexico (Plot 5, patch age 8 years)	Preserve area	31 species (0.063 ha, 1296 individuals per ha.)	Seed rain (all tree species): 32 (103.2%)<br>Seed rain (local tree species): 10 (32%)<br><br>Recruited-seedling (all tree species): 8 (25.8%)<br>Recruited-seedling (local tree species): 4 (12.9%)	N.A.	[4]

***** This study includes both tree and shrub species, N.A. = not applicable, all tree species = immigrant tree species and local tree species.

References

1. Ceccon, E.; Hernández, P. Seed rain dynamics following disturbance exclusion in a secondary tropical dry forest in Morelos, Mexico. *Rev. Biol. Trop.* **2009**, *57*, 257–269.
2. Grombone-Guaratini, M.T.; Alves, L.F.; Vinha, D.; Franco, G.A.D.C. Seed rain in areas with and without bamboo dominance within an urban fragment of the Atlantic Forest. *Acta Bot. Bras.* **2014**, *28*, 76–85, doi:10.1590/S0102-33062014000100008.
3. Reid, J.L.; Holl, K.D.; Zahawi, R.A. Seed dispersal limitations shift over time in tropical forest restoration. *Ecol. Appl.* **2015**, *25*, 1072–1082, doi:10.1890/14-1399.1.
4. Martinez-Ramos, M.; Soto-Castro, A. Seed rain and advanced regeneration in a tropical rain forest. *Vegetatio* **1993**, *107*, 299–318, doi:10.1007/BF00052231.

Table S8. Light intensity measured in this study during May, 2004 – January, 2005.

Month	Light intensity (x1,000 lux)
May	29.9
June	25.5
July	39.0
August	11.5
September	14.8
October	15.1
November	8.5
January	25.6

Figure S1 Abundances of seeds from 16 plant species detected in this experiment (A= *Bauhinia malabarica* Roxb., B= *Canarium subulatum* Guill., C= *Chromolaena odoratum* (L.) R.M.King&H.Rob, D= *Cratoxylum formosum* (Jack) Dyer, E= *Croton roxburghii* N.P. Balakr, F= *Dalbergia* sp., G= *Gardenia sootepensis* Hutch, H= *Garuga pinnata* Roxb, I= *Imperata cylindrica* (L.) P.Beauv, J= *Irvingia malayana* Oliv. ex A.W. Benn, K= *Lagerstroemia* (aff. venusta Wall. ex Cl., L= *Memecylon edule* Roxb, M= *Pennisetum polystachyon* Schult, N= *Pterocarpus macrocarpus* Kurz, O= *Shorea roxburghii* G.Don, P= *Terminalia triptera* Stapf)

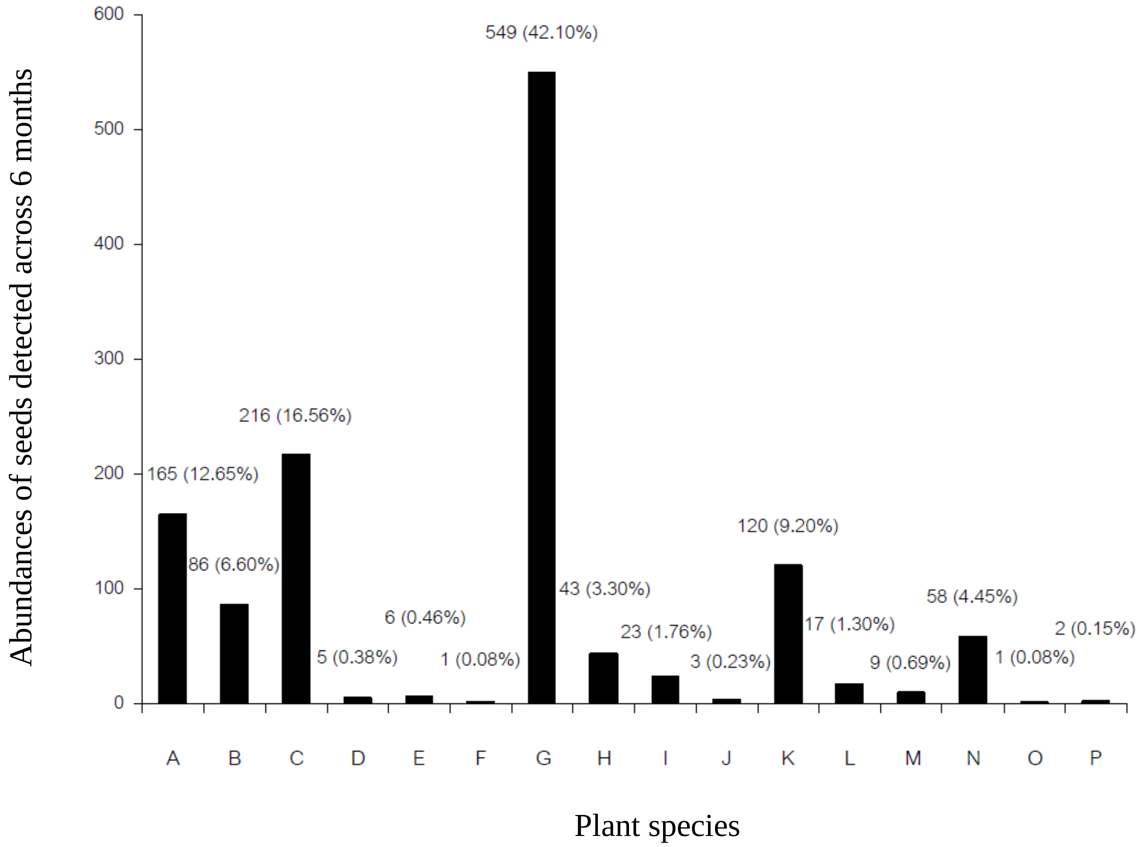

Supplement: Supplementary file 1 [file plants-09-01391-s001.pdf]
